# Quantification of the spatial distribution of primary tumors in the lung to develop new prognostic biomarkers for locally advanced NSCLC

**DOI:** 10.1038/s41598-021-00239-0

**Published:** 2021-10-22

**Authors:** Diem Vuong, Marta Bogowicz, Leonard Wee, Oliver Riesterer, Eugenia Vlaskou Badra, Louisa Abigail D’Cruz, Panagiotis Balermpas, Janita E. van Timmeren, Simon Burgermeister, André Dekker, Dirk De Ruysscher, Jan Unkelbach, Sandra Thierstein, Eric I. Eboulet, Solange Peters, Miklos Pless, Matthias Guckenberger, Stephanie Tanadini-Lang

**Affiliations:** 1 Department of Radiation Oncology, University Hospital Zurich and University of Zurich, Zurich, Switzerland; 2 Department of Radiation Oncology (MAASTRO), GROW School for Oncology and Developmental Biology, Maastricht University Medical Centre+, Maastricht, The Netherlands; 3 Center for Radiation-Oncology, KSA-KSB, Kantonsspital Aarau AG, Aarau, Switzerland; 4 Strahlentherapie und Onkologie, Universitätsklinikum Frankfurt, Frankfurt, Germany; 5 Swiss Group for Clinical Cancer Research (SAKK), Coordinating Center, Bern, Switzerland; 6 Department of Oncology, Centre Hospitalier Universitaire Vaudois (CHUV), Lausanne, Switzerland; 7 Department of Medical Oncology, Kantonsspital Winterthur, Winterthur, Switzerland

**Keywords:** Lung cancer, Tumour biomarkers, Translational research

## Abstract

The anatomical location and extent of primary lung tumors have shown prognostic value for overall survival (OS). However, its manual assessment is prone to interobserver variability. This study aims to use data driven identification of image characteristics for OS in locally advanced non-small cell lung cancer (NSCLC) patients. Five stage IIIA/IIIB NSCLC patient cohorts were retrospectively collected. Patients were treated either with radiochemotherapy (RCT): RCT1* (n = 107), RCT2 (n = 95), RCT3 (n = 37) or with surgery combined with radiotherapy or chemotherapy: S1* (n = 135), S2 (n = 55). Based on a deformable image registration (MIM Vista, 6.9.2.), an in-house developed software transferred each primary tumor to the CT scan of a reference patient while maintaining the original tumor shape. A frequency-weighted cumulative status map was created for both exploratory cohorts (indicated with an asterisk), where the spatial extent of the tumor was uni-labeled with 2 years OS. For the exploratory cohorts, a permutation test with random assignment of patient status was performed to identify regions with statistically significant worse OS, referred to as decreased survival areas (DSA). The minimal Euclidean distance between primary tumor to DSA was extracted from the independent cohorts (negative distance in case of overlap). To account for the tumor volume, the distance was scaled with the radius of the volume-equivalent sphere. For the S1 cohort, DSA were located at the right main bronchus whereas for the RCT1 cohort they further extended in cranio-caudal direction. In the independent cohorts, the model based on distance to DSA achieved performance: AUC_RCT2_ [95% CI] = 0.67 [0.55–0.78] and AUC_RCT3_ = 0.59 [0.39–0.79] for RCT patients, but showed bad performance for surgery cohort (AUC_S2_ = 0.52 [0.30–0.74]). Shorter distance to DSA was associated with worse outcome (p = 0.0074). In conclusion, this explanatory analysis quantifies the value of primary tumor location for OS prediction based on cumulative status maps. Shorter distance of primary tumor to a high-risk region was associated with worse prognosis in the RCT cohort.

## Introduction

Locally advanced non-small cell lung cancer (NSCLC) is the most advanced stage treated with curative intent to date, but patient outcome remains poor with a 5-year overall survival (OS) rate of approximately 5–35% despite multimodality treatment^1^.

In local treatments such as radiotherapy or surgery, the anatomical location of the primary tumor is of importance as anatomical obstacles may compromise treatment success. Primary tumors located either in the lower lobe^2,3^, more centrally^4^, or with chest wall invasion^5,6^ have been associated with poorer prognosis for different stages of NSCLC. However, these studies only associate tumor location with respect to predefined anatomic regions that only partially reflect the full 3D tumor location within the lung.

In recent years, more data is being collected, accelerating data mining approaches in cancer research. An excellent example to illustrate the additive value of data mining in cancer research is a study on the role of cardiac irradiation on OS of lung cancer patients^7^. Using a voxelized dosimetric comparison to identify regions of poor patient survival, they demonstrated that dose delivered at the base of the heart was more prognostic than previously used metrics^7^.

Here, we propose for the first time a data mining approach to investigate the association between tumor location and 2-year OS of locally advanced NSCLC patients. The extent of patient tumors is mapped to a reference patient anatomy and assigned with patient status. Thus, a given cohort can be represented by a voxelized spatial distribution of the cumulative status. This mapping approach has been previously used in brain lesions e.g. to study the impact of primary tumor entity on the spatial distribution of brain metastases^8,9^. In contrast to other studies, here the primary tumors are mapped while preserving the original shape of the primary tumors.

The aim of the study was two-fold. First, we identify areas in these maps with statistically significant decreased survival (DSA) to examine differences in outcome between surgical or radiochemotherapy (RCT) regimens. Second, the smallest distance of the primary tumor to the DSA is extracted to quantify the spatial distribution and perform outcome modeling on independent patient cohorts.

## Materials and methods

### Patient and imaging data

Computed tomography (CT) scans were collected retrospectively from five locally advanced stage IIIA/IIIB NSCLC cohorts (Table 1). Three patient cohorts were treated curatively with concurrent or sequential RCT (RCT1, RCT2, RCT3) and two with a combination of radiotherapy and chemotherapy and surgery (S1 and S2, more details can be found in the Supplement A). The RCT1 cohort is a publically available dataset that has been previously published^10–12^. The S1 cohort was collected from a multi-centric clinical trial (SAKK 16/00^13^), part of which the imaging data has been described elsewhere^14^. All remaining cohorts were based on single institution data.

**Table 1:** Overview of the stage III NSCLC patient cohorts used for this study.

Name	RCT1*	RCT2	RCT3	S1*	S2
Center	Maastro Clinic (LUNG1^10–12^)	Maastro Clinic (LUNG4)	Kantonsspital Aarau	Swiss multi-centric trial (SAKK 16/00^13^)	University Hospital Zurich
Patients	107	95	37	135	55
Treatment	RCT	RCT	RCT	RCT followed by surgery	RCT followed by surgery
OS events at 2 years	69.2%	50.5%	56.8%	37.8%	21.9%
Imaging	Single-institution	Single-institution	Single-institution	Multi-centric	Single-institution
In-plane resolution (mm)	0.98 (0)	0.98 (0)	0.98 (0)	0.98 (0.19)	1.04 (0.12)
Slice thickness (mm)	3.00 (0)	2.98 (0.15)	2.84 (1.04)	3.17 (1.18)	3.05 (0.45)
Number of CT reconstruction methods	8	6	2	16	6
Primary tumor volume (ml)	79.24 (94.4)	95.38 (102.12)	129.19 (124.60)	49.82 (56.81)	76.27 (99.44)
TNM edition	7	7	6	6	6

Cohorts marked with an asterisk were used to create the decreased survival areas. These cohorts are referred to as exploratory cohorts and the remaining cohorts as independent cohorts. Patients of the independent cohorts were used to extract the smallest distance to decreased survival areas as a potential prognostic factor. Values are reported with mean (standard deviation).

Radiation therapy planning CT scans were collected along with the contours from each institution in the RCT cohorts, whereas diagnostic CT scans were collected and contoured at our institution for the patients of the surgical cohorts.

The RCT1 collection has previously been approved for public release to The Cancer Imaging Archive. Only the publicly accessible dataset (known as NSCLC-RADIOMICS) has been accessed for this project. The RCT2 dataset is private at present; re-use of retrospective patient data from standard-of-care treatment has been approved by MAASTRO IRB. De-identified patient data was shared under terms and conditions of a bilateral legally executed data sharing agreement. For the RCT3, S1, and S2 cohorts, the data analysis was approved by the Swissethics and was carried out in accordance with Swissethics guidelines and regulations. All patients gave their informed general consent.

Cohorts RCT1 and S1 (indicated with an asterisk in Table 1) are referred to as exploratory cohorts, whereas the remaining cohorts are the independent cohorts. Based on the exploratory cohorts, maps of both spatial distribution and DSA are created. The independent cohorts will be used to extract the primary tumor distance to DSA and to test its prognostic value.

### Mapping of patient to reference

#### Reference patient

One head and neck cancer patient with two healthy lungs was selected as a reference patient frame. This patient had an age and body weight within 10% of the average patients in the S1 cohort. His patient characteristics were: male, 59 years, 65 kg, and 4710.28 ml lung volume (2544.42 ml right lung and 2165.86 left lung). The pre-treatment non-contrast CT scan had a resolution of 0.98 × 0.98 × 3.27 mm and was reconstructed with filtered-back projection and standard convolution kernel (GE Medical System, Discovery STE).

#### Deformable image registration

Due to the multi-centric setting of this study, image sets were heterogeneous in terms of acquisition and scanning settings (i.e., presence of contrast agents, different reconstruction kernels). Therefore, an intensity independent contour-only based deformable image registration was performed. The ipsilateral lung of each patient was registered using a deformable image registration to the reference lung (MIM Vista, v6.9.2.). As a first step, a new structure was created consisting of the ipsilateral lung, the primary tumor and present atelectasis or inflammation. In the second step, the patient CT along with this new structure served as a secondary image set and was registered deformably to the reference patient lung. For this purpose, a manual rigid registration of the main bronchus on the ipsilateral side was matched with the reference patient and set as a fixed landmark for the deformable image registration (REG Refine). In the third step, the deformation vector field (DVF) from the registration was extracted which contained displacement information for each deformation grid voxel.

#### Transfer of tumor to reference

In this study, we aimed to use the original shape of the primary tumor. The primary tumors were mapped to the reference patient using the DVF by first determining the center of mass in the patient frame followed by a coordinate transformation to the reference patient. The lung volumes differed in size between patients, therefore a sub-analysis was performed to study whether the ratio in volumes of tumor to lung correlated to survival at 2 years OS. Tumor-to-lung volumes differed significantly between S1 cohort patients with different outcome (Wilcoxon test, p < 0.007). Therefore, primary tumors were scaled isotropically to maintain the tumor to lung volume ratio within the reference patient. Implementation of primary tumor mapping as well as scaling were performed using VTK (v8.1.2.) and Python programming language (v3.7.1.). The transferred center of mass of the primary tumor from the in-house developed software agreed within ± 3 mm Euclidean distance with the MIM software within the S1 cohort.

### Map creation

For the two exploratory cohorts, two maps were created:

1. Frequency map representing anatomical locations of the primary tumors and
2. Frequency weighted cumulative status (fwCS) map where primary tumor location was uni-labeled with 2 years OS patient status (survival: 0 / death: 1).

Voxels which were covered by less than 2 patients were excluded from the analysis as they provided misleading information in fwCS maps. A more detailed description of the entire workflow can be found with the linked media (see https://radiomics-usz.github.io/lung_spatial_distribution/, accessed 12.10.2021).

### Identification of decreased survival areas and outcome prediction

**Figure 1:**
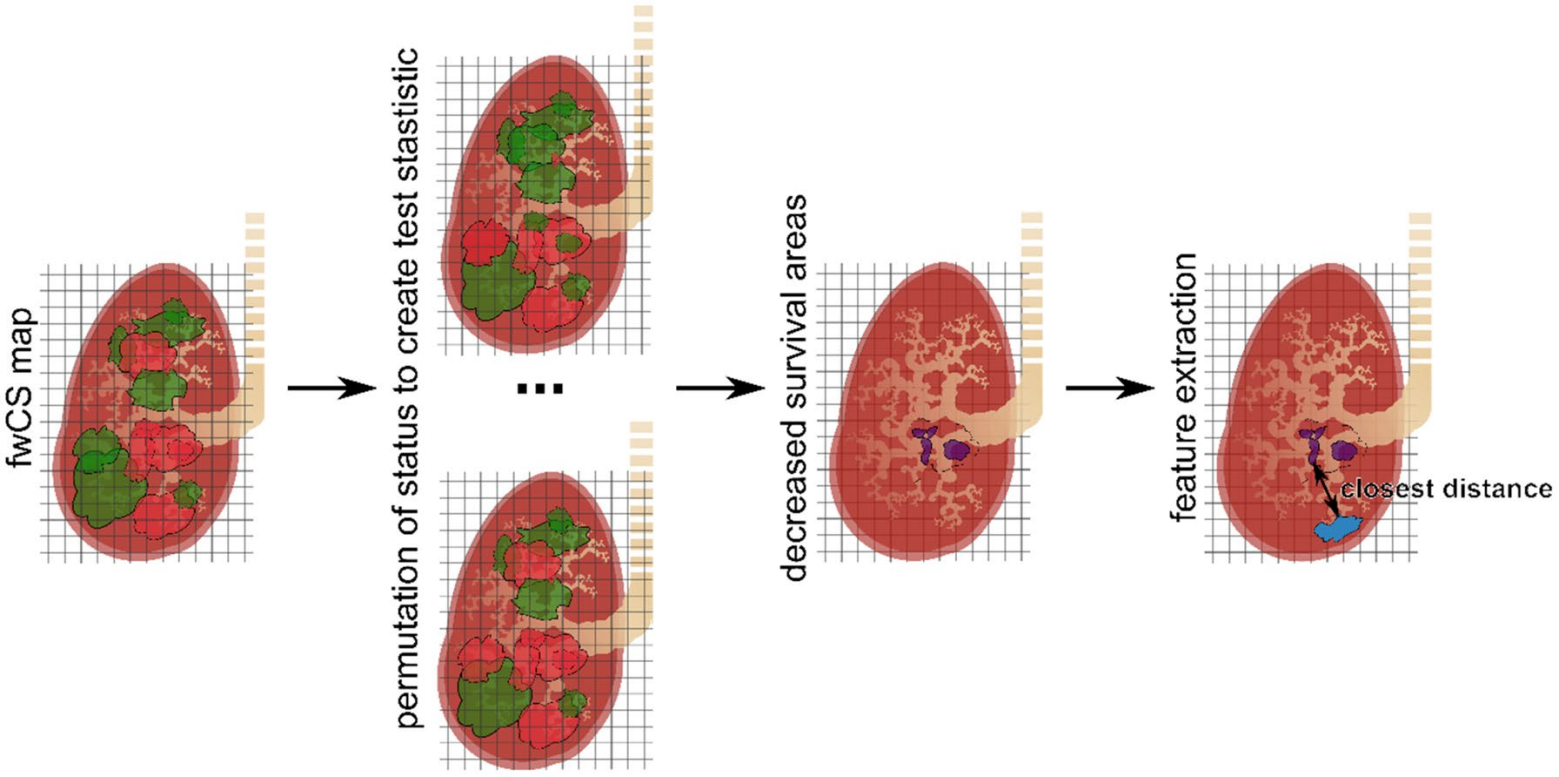
Identification of decreased survival areas (DSA) and extraction of the primary tumor’s closest distance. Based on the frequency weighted cumulative status (fwCS) map, a permutation test was performed to identify areas with statistically significant worse OS, from which the closest distance of a primary tumor (blue) was calculated.

For the exploratory cohorts, a permutation test was performed to identify areas with statistically significant decreased survival, which was adopted from a study comparing radiotherapy dose distributions^15^. Fig. 1 schematically shows the workflow: Given an fwCS status map of a patient cohort, a test statistic was created by calculating the ratio of mean $\mu$ and standard deviation $\sigma$ of each individual voxel. This test statistic serves as the null hypothesis. In each of the 500 repetitions $k$ with resampling, a new test statistic was calculated where the primary tumors were randomly assigned to survival or death. A voxel $i$ was associated with statistically significant worse outcome if ${\left(\frac{\mu }{\sigma }\right)}_{i}>95\% \; of \;{\left(\frac{\mu }{\sigma }\right)}_{k,i}$ (one-sided test).

Our hypothesis was that primary tumors closer to the DSA will have a worse prognosis, therefore the minimal Euclidean distance between primary tumor and DSA was computed for the remaining independent cohorts. Three scenarios were distinguished:

1. The tumor extent is outside any DSA, the smallest minimal distance is considered (positive).
2. The tumor extent touches DSA, distance is equal to 0.
3. The tumor overlaps with DSA, the largest minimal distance within the overlapping region is considered (negative).

The distance is further scaled with the radius of the tumor volume-equivalent sphere (distance/radius for scenario 1, and radius/distance for scenario 3). The rationale is to make the model tumor size independent, since large tumors will more likely have smaller positive distances or larger negative closest distances (see Supplement C). Finally, these distances were input of a logistic regression model and its performance was quantified with the area under the receiver operator characteristic (AUC) curve.

Further, we compared the performance of the model with models based on individual clinical parameters, such as T stage, tumor volume, left–right lung side. Since T and N stage definition is related to invasion and extent of the tumor into the carina, a sub-analysis was performed to test the distances against T and N stages using one-way ANOVA and Tukey’s test.

#### Post-processing

There are two post-processing steps performed to allow for a meaningful analysis. In the following two cases, patients were removed if:

1. the transferred tumor location did not match with the initial tumor location.
2. the patients did not overlap with the RCT1 or S1 maps.

The first is common when the primary tumor was in extreme superior inferior location. The second is to avoid primary tumors in regions that are not covered by the fwCS maps. We included only patients with a primary tumor overlap of 70% with the map, resulting in a total number of patients: RCT2 (n = 85), RCT3 (n = 32), S2 (n = 37).

## Results

### From fwCS map to decreased survival areas

**Figure 2:**
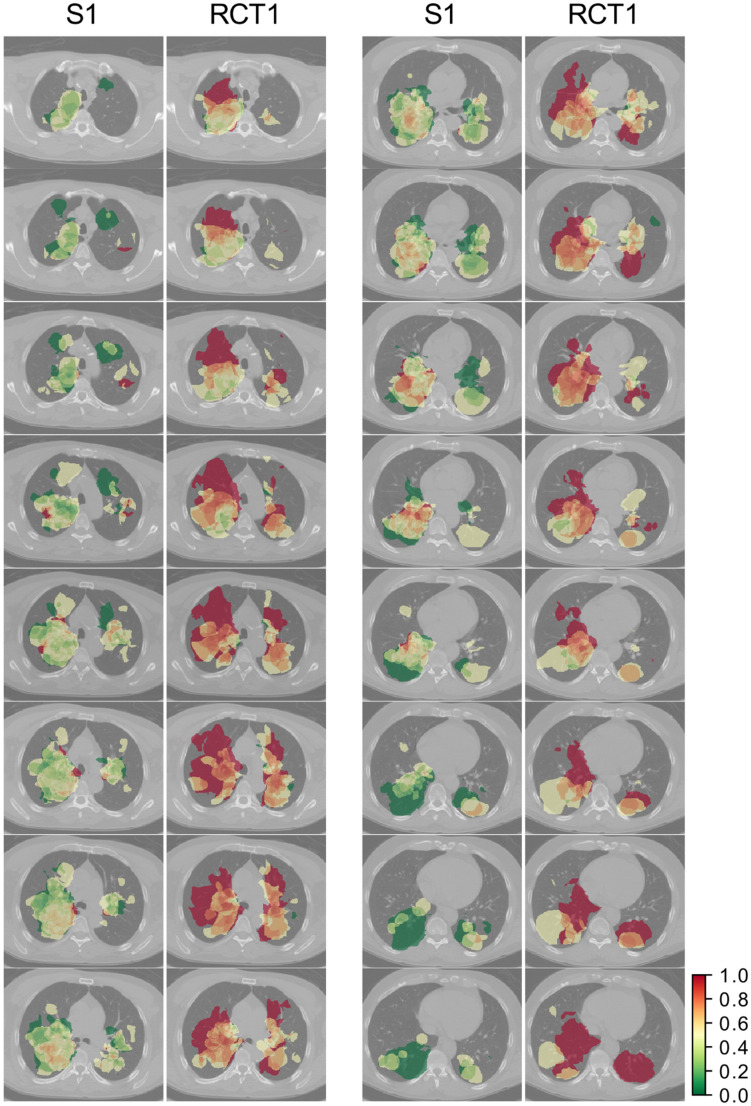
Comparison of frequency weighted cumulative status (fwCS) maps between S1 and RCT1 cohorts. Axial slices are shown with 3 slice step intervals (9.81 mm). The S1 cohort had fewer patients with an OS event at 2 years.

In Fig. 2, the fwCS maps are shown for S1 and RCT1 cohorts. Larger areas with worse prognosis were found in the RCT1 cohort compared to the S1 cohort. Primary tumors occurred mainly close to the mediastinum in both cohorts. Furthermore, primary tumors were frequently found in posterior position (the frequency maps can be viewed in the Supplement A). In total, 36.4% and 49.8% of right lung volume was covered in the S1 and RCT1 cohorts, respectively. The left lung coverage was lower compared to the right lung in both cohorts (18.28% and 27.29% for the S1 and RCT1 cohort, respectively). The difference in coverage between S1 and RCT1 is partially due to the smaller tumor volumes in S1 (Table 1).

**Figure 3:**
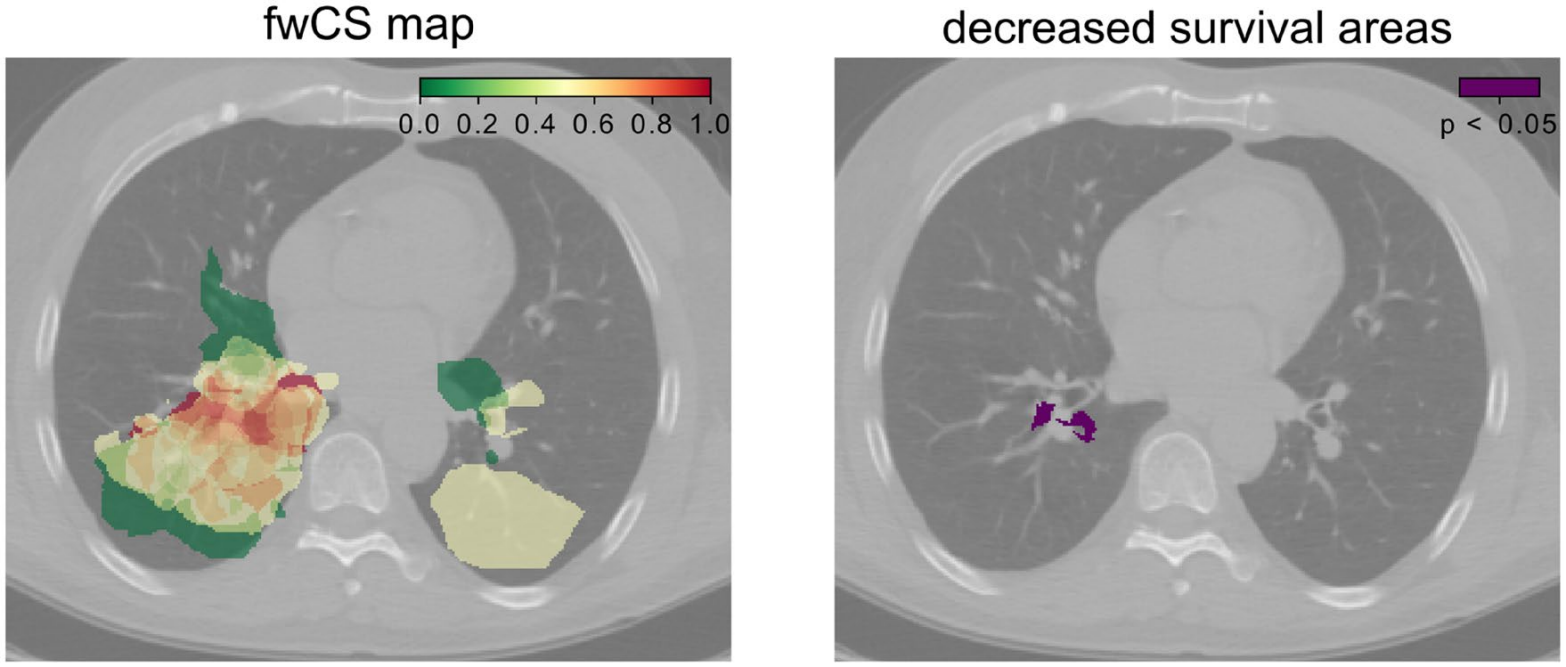
Axial CT slice of S1 frequency weighted cumulative status (fwCS) map on the left and decreased survival areas (DSA) labeled using the permutation method. Violet areas indicate statistically significant regions. Significant areas were found in the right lung close to the mediastinum.

For both S1 and RCT1 cohorts, areas with statistically significant large fwCS values could be found. An example of the fwCS map and corresponding decreased survival areas is shown in Fig. 3 for a patient in the S1 cohort. The areas with worse prognosis were found to be at the right lung side (Fig. 3).

### Comparison between treatment regimens

**Figure 4:**
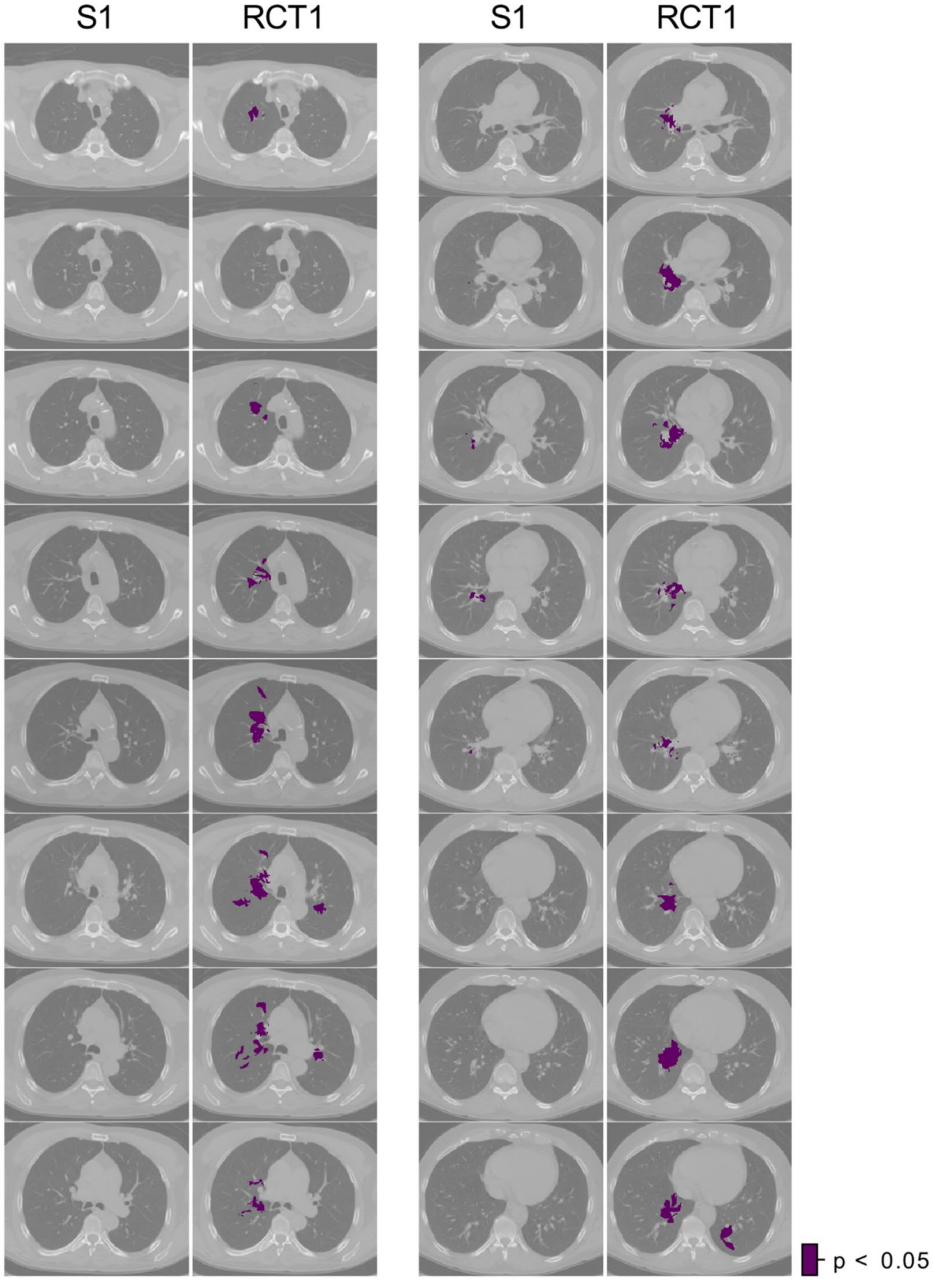
Comparison of decreased survival areas (DSA, violet) between S1 and RCT1 cohorts. Axial slices are shown with 3 slice step intervals (9.81 mm). S1 cohort shows an isolated location on the right lung side, whereas the DSA are spread in superior and inferior direction for RCT1.

A comparison of the DSA of the S1 and RCT1 cohorts showed only an isolated area at the right bronchi for the S1, whereas the DSA of the RCT1 further extended in cranio-caudal (CC) direction proximal to the mediastinum (Fig. 4).

### Outcome prediction

**Figure 5:**
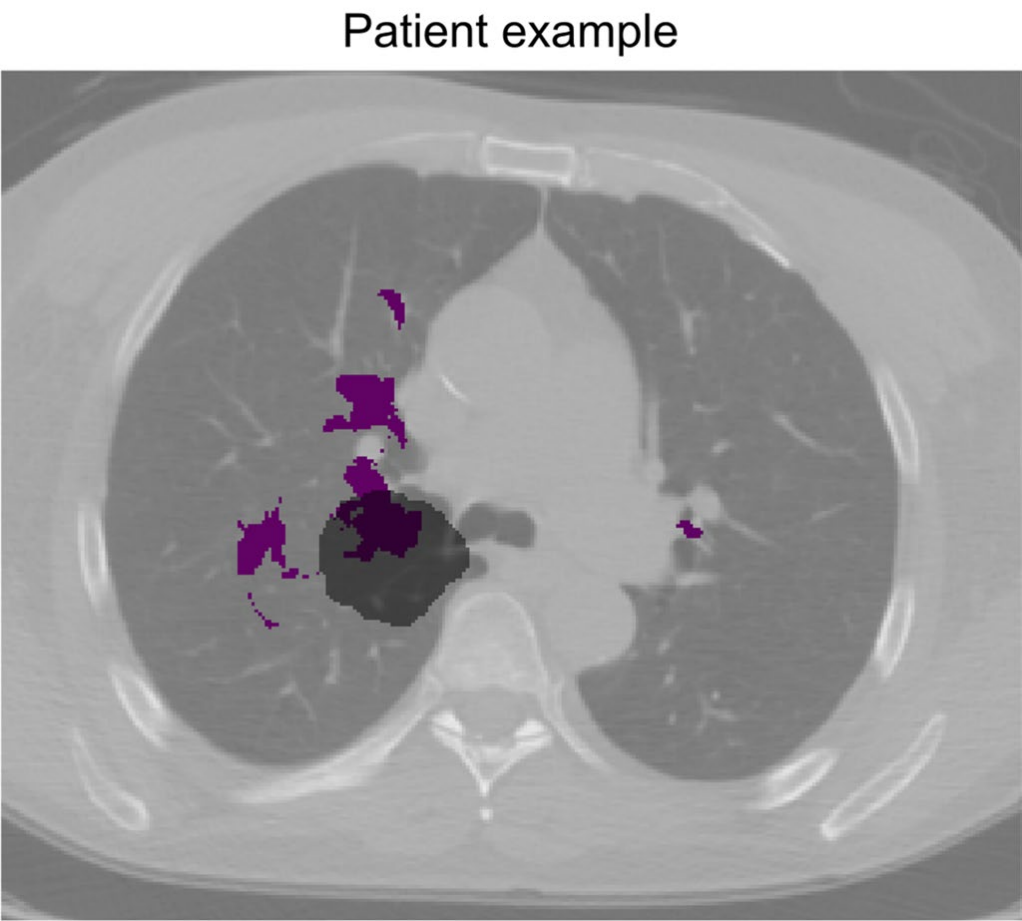
Example of an RCT2 patient with the primary tumor shown in gray and RCT1-decreased survival areas (DSA) shown in violet on an axial CT slice of the reference patient.

In Fig. 5, an example of RCT2 patient with its primary tumor overlapping with the DSA is shown. The performance of the model based on these distances for predicting 2 years OS were AUC_RCT2_ = 0.67 [95%CI: 0.55–0.78] and AUC_RCT3_ = 0.59 [0.39–0.79] for RCT patients, but showed bad performance for the surgery cohort (AUC_S2_ = 0.52 [0.30–0.74]). Smaller distance to DSA was associated with worse outcome (p = 0.0074, Mann–Whitney U test).

A statistically significant difference was observed when comparing the distances among T stage, but was not observed for N stages in the RCT2 cohort (one-way ANOVA, p = 0.001 and p = 0.256 for T and N stages, respectively). Only T1 stage tumors showed significantly different primary tumor distances compared to all other T stages (Tukey’s Test, Supplement C). T stage, tumor volume and left–right lung side showed worse performance compared to the smallest distance as in all cases the lower bound of the confidence interval was equal or lower than 0.5 (AUC = 0.51 [0.39–0.64], AUC = 0.62 [0.50–0.74] and AUC = 0.51 [0.38–0.61], respectively). A combined model had an AUC = 0.61 [0.49–0.73].

## Discussion

In this study, we introduced a data-driven voxelized cumulative status map approach to study the relationship between primary tumor location and 2 years OS in radically treated locally advanced stages IIIA/B NSCLC. Using a permutation test, we identified areas with statistical significantly worse prognosis and could show that these regions differ between locally advanced NSCLC patients treated either with RCT only or in combination with surgery. These regions were found mainly at the right side close to the mediastinum in both treatment regimens. However, these areas further spread in CC direction for RCT patients. In the second step, the smallest distance of the primary tumor to DSA were calculated on the independent patient cohorts. A logistic regression analysis showed that this distance performed acceptable in RCT patients but not for surgery patients. Smaller distance between tumor and DSA was associated with worse prognosis at 2 years OS.

Hypothesis driven studies investigating the impact of tumor location within the lung on patient outcome often focus on anatomical regions such as laterality (right/left), location within the lobes, centrality or chest wall invasion. The influence of tumor laterality in locally advanced NSCLC patients have shown inconclusive results. Right lung tumors were associated with significantly worse prognosis compared with the left side^16^, whereas other studies observed no statistical difference^17^ for patients treated with radiation therapy. Laterality was not found to be significant in resectable stage IIIA NSCLC patients^18^. More commonly, tumor location was studied in relation to lobe location. Primary tumors located in the lower lobe were significantly associated with higher mortality rate in 2,289 NSCLC patients of all stages and treated with curative intent or palliative intent with surgery, radiotherapy or chemotherapy (48.6% vs. 40.3%, *p* < 0.001)^19^. In locally advanced NSCLC, lower lung lobe locations have been associated with significantly poorer outcome compared to other lung lobe locations for patients treated with chemoradiotherapy^2^ as well as for patients treated with definitive radiation therapy^20^. For resectable stage IIIA NSCLC patients, contradicting results have been reported^3,19,21^. Proximity of lower lobe tumors favors to spread to the subcarinal station or contralateral hilar lymph nodes, causing in particular in advanced tumor stage a spread to central airway or mediastinum^3^. Due to limited diagnostic tools, lower lung lobe tumors are therefore difficult to stage and postoperative upstaging is often necessary^3^. Resectable NSCLC patients with multi-station lymph node involvement were found to have poorer outcome^22^. The association of tumor centrality with outcome is controversial due to the unclear definition^23–25^. Centrality is frequently defined as the one third of the hemithorax, where both, the concentric region from the hilum or sagittal planes from the central axis can be used^23^. A recent study investigated five definitions of centrality and showed no correlation with survival^24,25^. The tumor chest wall invasion infers challenges in correctly identifying the stage of the patient. Attachment to the chest wall was not consistently associated with prognosis for stage I NSCLC patients treated with SBRT^6,26^.

Our here proposed voxelized cumulative status maps, areas of worse prognosis could be identified in a quantitative 3D fashion. Areas associated with poor prognosis were found in more centrally located tumors in surgery (concentric region) and in RCT (sagittal plane definition). The RCT cohort had more areas with worse prognosis compared to surgical cohort, which may reflect the overall worse predisposition of inoperable patients. In the independent cohorts, primary tumors close to the DSA were observed to be associated with worse prognosis for RCT2 patients but not for RCT3. One possible explanation that the relationship could not be shown for the RCT3 may be because these patients originate from a different institution than the RCT1 on which the maps were created. As RCT1 and RCT2 cohort were from the same institution a more coherent patient selection and treatment was present compared to RCT3 cohort. Further, in RCT3 considerably fewer patients were involved and arguably inclusion of larger patient numbers could have improved the wide confidence intervals of the model. In a sub-analysis, a significant difference of primary tumor distances to DSA between T stages was observed. However, only T1 staged tumors had significant larger distances compared to other T stages. Models based on T stage, volume and laterality as well as their combination did not outperform the closest distance of the primary tumors to the DSA. No clear and distinct outcome association of tumor location was observed for surgically treated patients. Tumor location as a prognostic factor is regarded controversial also due to unknown underlying mediating factors such as histology or possibly mutation status differences between patient groups^17,19,27^. Further studies are needed to interpret the origin of the differences in spatial distribution. Due to the lack of biological data of the patient cohorts, this was beyond the scope of this study.

The number of patients in this study was limited and more patients would be needed to cover the entire lung, however our initial promising results may facilitate further multi-institution data collection. The permutation test proved feasible to identify areas with decreased survival taking into account multiple testing. Furthermore it allowed to account for variability in data entry per voxel. Also, the limited numbers in patients hindered a stratified analysis by therapy regimens (concurrent/sequential therapy or chemotherapy/radiochemotherapy prior to surgery). Additionally, the model performances likely would improve if only diagnostic CT scans were collected. This was the case in the surgery cohorts, however in a sequential RCT treatment, chemotherapy might have influenced the anatomy of the primary tumors and thus the distances. We however had only a small number of patients with sequential RCT. Due to the retrospective nature of this study, patients received different chemotherapy and radiation therapy regimens, which was not possible to account for. Therefore, a prospective study of the analysis would be highly desirable. Nevertheless, RCT1 and RCT2 being from the same institution and same treatment era, our methodology showed feasibility to quantify the tumor location as a prognostic factor. However, further analysis are needed to be able to compare and confirm the relative performance of the model based on the distances with clinical parameters on larger datasets. The results presented may be influenced by the reference patient selected. In this analysis, we used a reference patient who was matched for age, sex, lung volume, and appropriate imaging characteristics with 10% patient variability. Nevertheless, results may differ if other reference patients are used. Therefore future studies should utilize the same reference patient as presented in this study similar to a patient lung anatomy atlas. The accuracy of the mapping of the primary tumors to the reference is influenced by the accuracy of the deformable image registration and therefore can influence the outcome prediction. However when we compared the center of mass measures of the in-house developed software and the MIM software the deviation was within an acceptable 3 mm in all directions, allowing a robust outcome prediction. Since the shape of the lung can vary across a patient cohort, the mapping of the primary tumor center of mass can be misplaced. Therefore, lung tumors positioned in extreme positions (inferior or superior position) should be excluded from the analysis. Since those extreme locations were not found frequent, they were automatically discarded by the permutation test. Further rotation of the tumors from the patient to reference frame were not accounted for. Lastly, respiratory motion can exhibit different degree of blurring depending on the location of the tumor within the lung potentially resulting in stronger blurring effects in more inferior positions possibly affecting the spatial tumor extent of the primary tumors. One option to account for motion related artefacts would be to extend the tumor shape by a motion related margin, however since this work was a retrospective study, we could not further collect motion-related measurements.

## Conclusion

This data mining approach, based on voxelized cumulative status maps, showed promising results in quantifying the value of primary tumor location for overall survival prediction. Smaller distance of primary tumor to a high-risk region was associated with worse prognosis in the RCT cohort.

## Supplementary Information

Supplementary Information.
