# Supplementary material for: Quantification of the spatial distribution of primary tumors in the lung to develop new prognostic biomarkers for locally advanced NSCLC
Source: Sci Rep. 2021 Oct 22;11:20890. doi: 10.1038/s41598-021-00239-0 (PMC8536672; doi:10.1038/s41598-021-00239-0)
Supplement: Supplementary file 1 — Supplementary Information. [file 41598_2021_239_MOESM1_ESM.pdf]

Quantification of the spatial distribution of primary tumors in the lung to develop new prognostic biomarkers for locally advanced NSCLC

Vuong D[1]*, Bogowicz M[1], Wee L[2], Riesterer O[1,3], Vlaskou Badra E[1], D'Cruz L[4], Balermpas P[1], van Timmeren JE[1], Burgermeister S[1], Dekker A[2], De Ruysscher D[2], Unkelbach J[1], Thierstein S[5], Eboulet E[5], Peters S[6], Pless M[7], Guckenberger M[1], Tanadini-Lang S[1]

[1] Department of Radiation Oncology, University Hospital Zurich and University of Zurich, Zurich, Switzerland
[2] Department of Radiation Oncology (MAASTRO), GROW School for Oncology and Developmental Biology, Maastricht University Medical Centre+, Maastricht, The Netherlands
[3]Center for Radiation-Oncology KSA-KSB, Kantonsspital Aarau AG, Aarau, Switzerland;
[4]Strahlentherapie und Onkologie, Universitätsklinikum Frankfurt, Frankfurt, Germany;
[5]Swiss Group for Clinical Cancer Research (SAKK), Coordinating Center, Bern, Switzerland;
[6]Department of Oncology, Centre Hospitalier Universitaire Vaudois (CHUV), Lausanne, Switzerland;
[7]Department of Medical Oncology, Kantonsspital Winterthur, Winterthur, Switzerland

Supplement A: Patient characteristics

	RCT1	RCT2	RCT3	S1	S2
**Number of Patients**	107	95	37	135	55
**Sex**					
Female	39	37	13	48	26
Male	68	58	24	87	29
**Age** (Median (IQR))	65.5 (42.6)	68 (47)	66 (43)	60 (39)	67 (33)
**Tumor Histology**					
Adenocarcinoma	13	*	16	58	17
Squamous cell carcinoma	38	*	16	43	3
Large cell carcinoma	56	*		8	
Poorly diff./Unknown		*		26	3
**Tumor Stage**					
T1	12	8	2	21	5
T2	44	28	13	78	23
T3	8	18	11	34	12
T4	43	41	11	2	15
**Nodal Stage**					
N0	14	7	3		5
N1	3	4	2	1	8
N2	56	46	14	134	41
N3	34	37	18		1
**Metastasis Stage**					
M0	105	95	32	132	54
M1			4	3	1
M2	2				
Unknown			1		
**Treatment**					
Concurrent RCT	*	86	32		2
Sequential RCT	*	9	5	70	6
Adj. Chemotherapy Therapy	*				6
Chemotherapy	*			65	41
**Survival Data**					
OS events at 2 years	69.2%	50.5%	56.8%	37.8%	69.2%

Table 1: Patient characteristics. Asterisks indicate insufficiently reported data.

Supplement B: Frequency maps

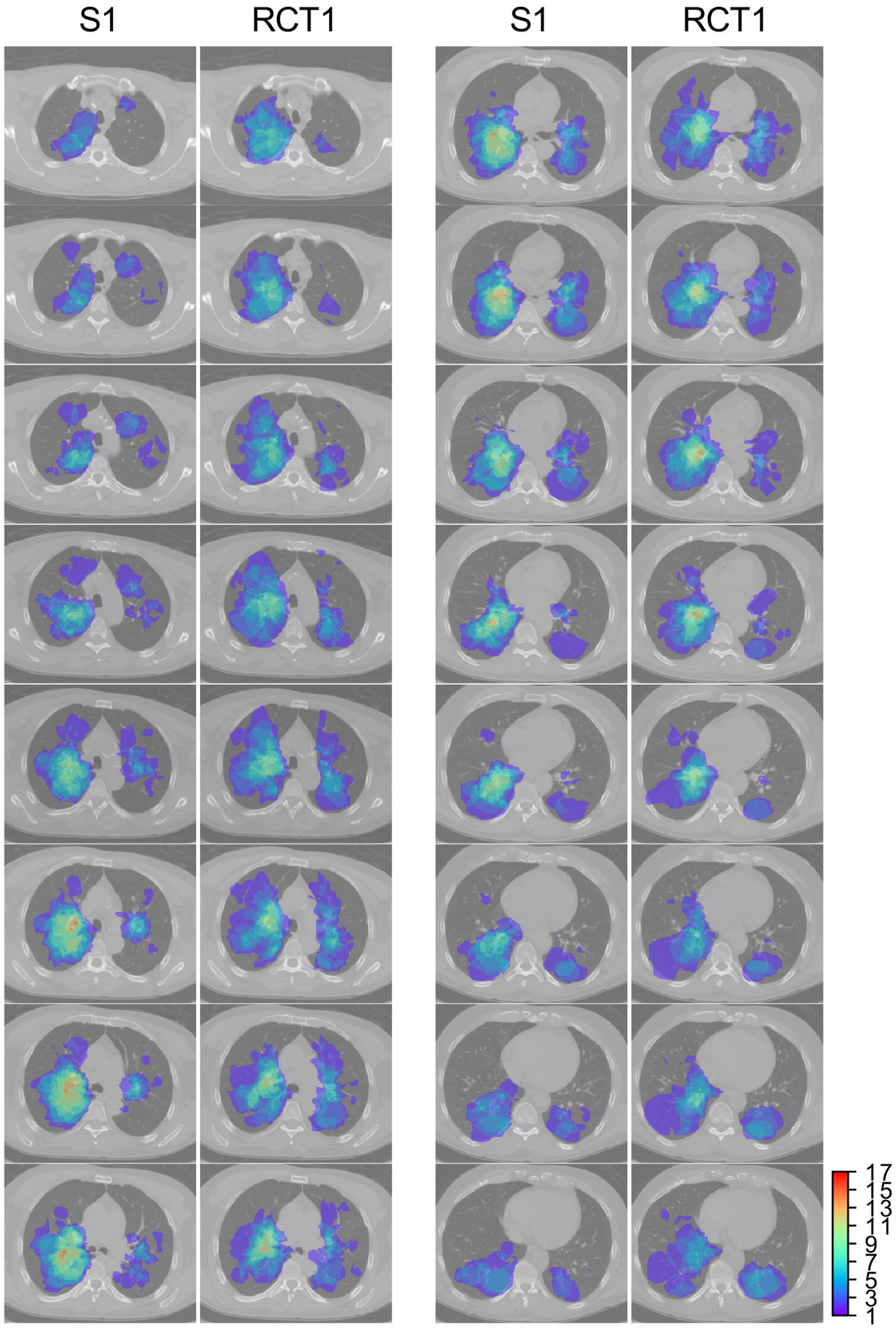

Figure 1: Comparison of frequency maps between S1 and RCT1 cohorts. Axial slices are shown with 3 slice step intervals (9.81 mm).

Supplement C: Correlation of primary tumor distance with volume, T and N stage

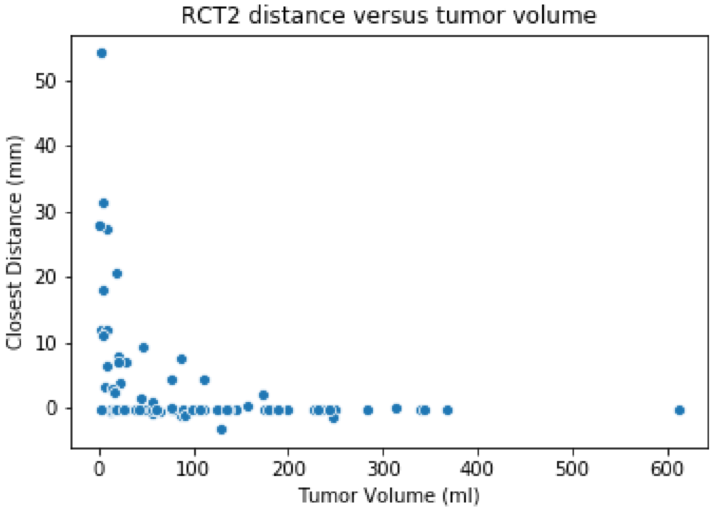

Figure 2: Scatterplot of distances and tumor volume for RCT2 cohort.

In a sub-analysis we performed a one-way ANOVA test to study the difference in distance between primary tumor distance and decreased survival areas for different T and N stages. For the T stages, the average distance was 14.48 mm (±8.88), 1.67 mm (±5.69), 4.5 mm (±13.18) and 1.27 mm (±5.54) for increasing T stage. There was a statistically significant difference between the primary tumor distances of different the T stage (F= 5.88, p-value= 0.001). The distances of T1 stage tumors were significantly different any other T stage (p-values=0.002, 0.0319, 0.001 for T2, T3 and T4, respectively, Tukey test).

For N stage, the closest tumor-to-DSA distance was 0.19 mm (±1.51), -0.23 mm (±0.12), 2.13 mm (±6.35), 5.34 mm (±11.42) for increasing N stage. No statistically difference was found between the primary tumor PT distances of different the N stages (F=1.37, p-value=0.256).
